# Muskoskeletal manifestations of mild form of mucopolysaccharidosis iva - a clinical case

**DOI:** 10.1186/1546-0096-12-S1-P163

**Published:** 2014-09-17

**Authors:** Margarita Ganeva, Stefan Stefanov, Daniela Avdjieva, Radka Tincheva, Ivanka Sinigerska, Dinko Zahariev, Julia Bachvarova, Zdravka Todorova

**Affiliations:** 1 Rheumatology, University Children’s Hospital, Sofia, Bulgaria; 2 Endocrinology and Genetics, University Children's Hospital, Sofia, Bulgaria; 3 National Genetic Laboratory, University Obstetrics and Gynecology Hospital “Maichin Dom”, Sofia, Bulgaria; 4 Radiology, University Children’s Hospital, Sofia, Bulgaria; 5 Endocrinology and Genetics, University Children’s Hospital, Sofia, Bulgaria

## Introduction

Mucopolysaccharidosis IVA (MPS IVA) is a rare inherited metabolic disorder caused by galactosamine-6-sulfate sulfatase (GALNS) enzyme deficiency that leads to progressive lysosomal accumulation of glycosaminoglycans (GAGs). MPS IVA has a variable age of onset and variable severity. Clinical presentation is heterogeneous, and some cases are only mildly affected. Key clinical features include short stature, skeletal dysplasia, dental anomalies, and corneal clouding. It is the milder forms of MPS that are often diagnosed late or misdiagnosed as an inflammatory joint disease.

## Objectives

To describe a clinical case of muskoskeletal involvement in a patient with MPS IVA and to raise awareness for the timely diagnosis.

## Methods

Case report.

## Results

We present a case of a 12-year old boy with a 2-year history of hip and lower back pain, morning stiffness and disordered gait. A CT scan was performed prior to admission – it revealed changes in the femoral heads as a result of severe narrowing of the epiphyseal region, shallow acetabulum and femoral neck shortening.

On admission the patient had hip pain and limited hip internal and external rotation, as well as lower back pain that worsens when bending, scoliosis, knee and ankle pain, inability to squat, abnormal gait. The boy had normal stature. Due to X-ray changes compatible with coxa plana, investigations to rule out MPS were made. The patient did not have corneal clouding. A thoracic and lumbar spine X-ray was performed - the observed changes were suggestive of MPS.

Urine and blood specimens were referred to the National Genetic Laboratory. The patient was found to have elevated GAG levels in his urine. Subsequent enzyme analysis revealed reduced GALNS enzyme activity, and the diagnosis of MPS IVA was made.

## Conclusion

The boy was referred to the rheumatologist due to hip and lower back pain. The diagnosis of the classical forms of MPS is relatively easy due to the presence of typical clinical features. Diagnostic delays occur frequently in patients with mild forms of MPS which has an impact on the institution of appropriate therapy as early as possible.

## Disclosure of interest

None declared.

